# The role of the TIM-3 receptor in inflammatory bowel disease and autoimmunity: implications for TIM-3 and TCR crosstalk

**DOI:** 10.1007/s00109-025-02631-z

**Published:** 2026-01-02

**Authors:** M. Gabel, A. Knauss, M. F. Neurath, B. Weigmann

**Affiliations:** 1 Department of Medicine 1, University of Erlangen-Nuremberg, Kussmaul Campus for Medical Research, Kussmaul-Forschungscampus, Hartmannstr. 14, 91054 Erlangen, Germany; 2 Deutsches Zentrum Immuntherapie (DZI), Erlangen, Germany; 3 FAU Profile Center Immunomedicine, Erlangen, Germany

**Keywords:** TIM-3, T cell receptor, TCR-signaling, Inflammatory bowel disease, Inflammation, Autoimmune disease

## Abstract

The T cell immunoglobulin and mucin-domain receptor 3 (TIM-3) is an immune checkpoint receptor with complex effects on T cell activation and tolerance. TIM-3 is expressed on the surface of T cells in close proximity to the T cell receptor (TCR), where it mainly acts co-inhibitory, despite lacking typical inhibitory immunoreceptor inhibition motifs, to regulate T cell activity or apoptosis. However, the underlying molecular mechanisms of its intracellular signal transduction remains incompletely understood. Central for the function of TIM-3 are conserved cytoplasmic tyrosine structures that require phosphorylation for downstream signaling. Previous findings suggest that several kinases linked to TCR signaling, including the Src family kinase Lck and the Tec kinase ITK can interact with TIM-3 and thereby regulate TCR signaling and fine-tune T cell activation and tolerance. The ligand-dependent phosphorylation of TIM-3 by ITK, together with the displacement of the adapter protein Bat3, illustrates how TIM-3 signaling modulates early TCR activation. In this review, we describe the interactions of TIM-3 and its various actors that can trigger TCR signaling. This review also aims to shed light on this complex network by providing an overview of the immune checkpoint receptor TIM-3 and its influence on various autoimmune diseases. The focus here is clearly on inflammatory bowel disease (IBD), and open questions about the TIM-3 network are outlined.

## Introduction

T cells are essential players of the adaptive immune system providing effective immune responses [1, 2]. Upon immune stimulation, antigen-presenting cells facilitate T cell activation by presenting foreign antigens on their surface, to which the T cell receptor (TCR) can bind. The subsequent signal is transmitted into the cell via a complex biochemical process involving molecular changes, with the participation of several proteins and kinases at the cell membrane, but also in the cytoplasm. Downstream TCR signaling cascades result in the activation of transcription factors that initiate the transcription and translation of genes into proteins which enables T cell effector functions and differentiation processes [3, 4]. Additional receptors on the T cell surface can have a co-stimulatory or co-inhibitory effect on TCR signaling, thereby modulating intracellular signal transduction and amplifying or dampening T cell activity [5]. When excessive or chronic antigen stimulation occurs, co-inhibitory receptors are increasingly expressed on T cells, which in turn dampen excessive T cell activity and the associated damage caused by overactive T cell responses [6]. The T cell immunoglobulin and mucin-domain receptor 3 (TIM-3) is a known immune checkpoint receptor with identified co-inhibitory functions [7]. Recently, this receptor attracted great interest not only in context with cancer but also due to its involvement in the pathogenesis of autoimmune diseases and particularly in inflammatory bowel disease (IBD) [7–10]. Early studies established a well-known role of TIM-3 in mediating apoptosis of T cells, especially T_H_1 cells, upon binding to its ligand galectin-9 making TIM-3 popular for dampening T_H_1 immune responses [11]. Nevertheless, individual studies attribute a dual role for TIM-3 and also describe co-stimulatory functions of TIM-3, which often leads to discussions about if TIM-3 is a co-inhibitory or co-stimulatory receptor [12]. These uncertainties originate partially from specific structural features of the TIM-3 receptor. The TIM-3 receptor has an immunoglobulin variable domain, a mucin domain, a transmembrane domain, and an intracellular cytoplasmic tail. Unlike other better-studied immune checkpoint receptors, such as PD-1, it has no known inhibitory immunoreceptor tyrosine-based inhibition motifs (ITIMs) in the region of its cytoplasmic tail [13]. However, TIM-3 has six tyrosine residues within its cytoplasmic tail, of which two tyrosines can be phosphorylated for downstream signaling [14–16]. Although several studies have attempted to investigate the function of TIM-3 and its precise signaling, many questions remain unanswered, particularly in relation to its interaction with the T cell receptor downstream cascade. The effects and functions of TIM-3 are highly dependent on the phosphorylation of its cytoplasmic tyrosine structures, which can be mediated by several TCR-related kinases [15]. This review aims to summarize current findings in TIM-3 signaling, its interconnectivity to TCR signaling, and its linkage to autoimmune disorders with an emphasis on IBD. Furthermore, key players of TIM-3’s intracellular phosphorylation will be highlighted and gaps in knowledge identified raising open questions for future research.

## Characteristics of the TIM-3 molecule and its ligands

The gene of TIM-3 is termed Hepatitis A virus cellular receptor 2 (*HAVCR2*) in humans and *Havcr2* in mice, respectively [17]. *HAVCR2* RNA expression shows high levels in the human brain, kidney and urinary bladder, as well as in hematopoietic and lymphoid tissues, particularly in lymph nodes and spleen (data obtained from the human protein atlas: https://www.proteinatlas.org [18]). Lymphoid tissues exhibit higher TIM-3 protein expression compared to most other organ tissues. Comparable TIM-3 protein expression is observed in the gastrointestinal tract, whereas the highest TIM-3 protein expression is detected in the kidney [18]. RNA single cell data specifically identified NK cells, macrophages, monocytes, and granulocytes as relevant immune cells expressing *HAVCR2*. However, in colon tissue T cells display the highest *HAVCR2* transcript [18]. Pioneering work from Monney et al., identified and characterized the TIM-3 protein as a T_H_1-specific cell surface protein consisting of 281 amino acids in mice and 301 amino acids in humans [7]. They further detected a 63% amino acid identity between the human and murine protein and located their genes on the human chromosome 5.q33 and mouse chromosome 11B1 [7]. The protein is composed of an immunoglobulin variable-region-like domain and a mucin domain consisting of serine and threonine residues by 31%. Both domains represent the extracellular compound of the protein providing sites for both O-linked and N-linked glycosylation. The extracellular domains are followed by a transmembrane domain and an intracellular region containing 6 tyrosine residues [7, 19]. In mice the residues Y256 (corresponding to Y265 in human TIM-3 receptor) and Y263 (corresponding to Y272 in human TIM-3 receptor) are reported to be of special interest as they enable interactions with the human leukocyte antigen B (HLA-B) associated transcript 3 (Bat3) [14, 20], more on this later. In 2007, Cao et al. identified and described unique structural features of the murine TIM-3 protein. Its IgV domain varies from other immunoglobulin superfamily members as it shapes a so-called FG-CC cleft. Two additional intramolecular disulfide bonds are formed by four noncanonical cysteines resulting in close proximity of the CC’ loop to the FG’ loop that are located across each other in classical IgV domains [21].

At present, four ligands have been discovered for the TIM-3 proteins namely galectin-9 (Gal-9), phosphatidylserine (PtdSer), the high-mobility group box 1 (HMGB1) as well as the carcinoembyrotic antigen-related cell adhesion molecule 1 (CEACAM1). They are presented in the following section.

**Gal-9** (encoded by the *LGALS9* gene) was the first TIM-3-ligand identified by Zhu et al. [22]. It is a carbohydrate-recognizing protein, belongs to the galectin family, also known as S-type lectins, can be found in both humans and mice, and has a molecular mass of 30–35 kD [23]. Gal-9 is widely expressed in the organ systems and by various cell types. High protein levels can be found including but not limited to the brain, lung, and gastrointestinal tract whereas RNA levels are highly increased in the blood [24]. Single cell analysis identified enterocytes, paneth cells and goblet cells as major cell types expressing *LGALS9* [18]. Upon binding to the extracellular TIM-3 glycosylation sites, Gal-9 can provoke T_H_1 cell death in vitro within 4–8 h associated with a distinct calcium flux; no such effect was detected in T_H_2 cells [11]. However, apoptosis of T_H_1 cells was not fully eradicated in TIM-3-deficient cells proposing that Gal-9 may mediate cell death by binding additional receptors. Zhu et al. further reported that Gal-9 can eliminate IFN-γ producing CD4^+^T cells and reviewed that Gal-9 expression can be stimulated by IFN-γ and IL-1β. This suggests a negative feedback loop in which IFN- γ can not only promote inflammatory processes but can also induce inhibitory Gal-9 counteracting and dampening inflammation by targeting T_H_1 cells via TIM-3 [11].

**PtdSer** is a phospholipid and part of plasma membranes [25]. PtdSer binds to the structural pocket formed by the CC and FG loops of the TIM-3 protein and all other proteins of the TIM family except for murine TIM-2 [26]. PtdSer is a marker designating cells that undergo apoptosis. In healthy cells, it is localised within the plasma membrane but it is exposed on the cell surface of apoptotic cells in order to be recognized by phagocytes [27]. Fibroblasts and antigen-presenting cells such as dendritic cells can bind to PtdSer-expressing apoptotic cells via TIM-3 and can mediate their phagocytosis. T cells associated with the TIM-3 receptor are also able to sense PtdSer and interact with apoptotic cells, however, they do not contribute to their clearance [28].

**HMGB1** (encoded by the *HMGB1* gene) is a plasma protein showing a low cell type specificity as it is expressed in multiple cells and tissues in humans [18]. It is involved in various cellular processes, influences and activates both innate and adaptive immune responses that are mediated by sensors of nucleic acids [29, 30]. HMGB1 is reported to interact with TIM-3 on dendritic cells associated with tumor microenvironments and it is presumed that TIM-3 could negatively regulate innate immune responses promoted by HMGB1 and triggered by nucleic acids. It was further reported that the HMGB1/TIM-3 complex can diminish T cell responses and T cell proliferation [31].

**CEACAM1** (encoded by the *CEACAM1* gene) is highly expressed in the human gastrointestinal tract on RNA and protein levels followed by the human bone marrow and lymphoid tissues [18]. RNA single cell type specificity identified mainly distal enterocytes and Paneth cells that express CEACAM1 [18]. It appears mainly in context of infectious diseases, autoimmunity and has a tumor-associated function especially in colorectal cancer [32]. It is known as an inhibitory factor on T cells by modulating the activation threshold of T cells [33]. However, it can also be co-expressed together with TIM-3, where it shows tolerance induction of T cells [16]. CEACAM1 can bind to TIM-3 receptors [16]. Subsequently, intracellular processes are stimulated leading to inhibited immune responses that dampen anti-tumor signaling [13].

## Role of the TIM-3 receptor in autoimmune and inflammatory bowel diseases

After the structural characterization of TIM-3 and the identification of its ligands, attention is directed to its role in autoimmune diseases, with a focus on IBD. The effects of TIM-3 absence or dysfunction are noteworthy. Genetic alterations of *HAVCR2,* as reported and identified by Gayden et al. and Polprasert et al. were associated with a TIM-3 deficiency, resulting in uncontrolled immune activation and the increased production of pro-inflammatory cytokines, such as TNF-α. This was attributed to impaired TIM-3 surface expression resulting from protein misfolding [34, 35]. Another study by Zhang et al., investigated the relationship between *HAVCR2* polymorphisms and the risk of autoimmune diseases and identified the TIM-3+4259A>C polymorphism to be associated with rheumatoid arthritis [36]. In another study, Baldrich et al. also showed that the absence of TIM-3 directly influences the development of IBD. They delineated the clinical profile of a patient who developed IBD after allogeneic stem cell transplantation. Expression of TIM-3 was minimal on immune cells within the patient’s intestinal mucosa. Whole exome sequencing and Sanger sequencing of the donor-derived DNA unveiled the presence of a rare *HAVCR2* missense mutation, denoted cA291G; p.I97M, which resulted in the aberrant folding of TIM-3 and the loss of its surface expression [10]. Overall, the results from genetic modifications of *HAVCR2* show that impaired TIM-3 function leads to dysregulated immune activation and increased proinflammatory cytokines, which is also frequently observed in autoimmune diseases.

Furthermore, TIM-3 dysregulations have been implicated in the pathophysiology of IBD. Increased TIM-3 mRNA expression levels were detected by Kim et al. in the colonic mucosa from patients with Crohn’s disease (CD) compared to healthy controls. The same group reported a positive correlation of the severity of inflammation with the expression of *HAVCR2*, and that elevated TIM-3 expression could be downregulated by the treatment with infliximab [37]*.* Studying PBMCs and colon tissue samples from patients with ulcerative colitis (UC) or mice with DSS-induced colitis, Shi et al., detected a dysregulation of TIM-3 compared to controls [38]. Contrary to Kim et al. TIM-3 transcript was significantly diminished in colon samples from UC patients and colitis mice. Additionally, enhanced T_H_17 cell function was observed in UC patients and Shi et al., proposed that the increased T_H_17 cell activity might be the consequence of downregulated TIM-3 expression [38]. To explore potential interventions for TIM-3 dysregulation, Shi et al. evaluated the effects of antagonistic α-TIM-3 antibodies and recombinant Gal-9 in murine models of colitis. The Injection of α-TIM-3 antibodies resulted in exacerbated tissue damage and enhanced transcript and protein levels of the proinflammatory cytokines IL-17 and IFN-γ, whereas Foxp3 mRNA levels were alleviated. Moreover, treatment with the TIM-3 ligand Gal-9 improved colitis progression, decreased weight loss and diminished the expression of IL-17 and IFN-γ [38]. The impact of blocking TIM-3 in vivo was also tested by Li et al. in a TNBS-induced colitis model. They detected a significantly impaired disease outcome marked by increased weight loss, mortality, histological evaluated tissue damage and elevated expression of pro-inflammatory cytokines. In further analysis, they reported a decreased percentage and number of CD4^+^Foxp3^+^ Tregs. Surprisingly, blocking TIM-3/Gal-9 signaling did not affect apoptosis of CD4^+^ cells significantly [39]. Shi et al., and Li et al., suggested that the negative impact of theTIM-3 blockade mostly results from enhanced T_H_17 function and attenuated Treg cell response [38, 39]. This goes in line with data from Gautron et al., as they showed that TIM-3^+^ Tregs cells can specifically inhibit T_H_17 cells, an ability that Tregs lacking TIM-3 cannot. Stimulating TIM-3 might enhance the suppressor function of regulatory T cells while blocking results in the opposite effect [40]. A recent study from Xiong et al., focused on the effect of the TIM-3 ligand Gal-9 in induced colitis in mice. Administration of Gal-9 led to overall ameliorated disease severity in TNBS-induced colitis but not DSS-induced colitis. Furthermore, decreased serum levels of IFN-γ, IL1-β, and IL-6 were detected [41]. Overall, the data indicate that TIM-3 blockade promotes IBD severity, whereas TIM-3 activation improves symptoms, with multiple T cell subsets contributing to these TIM-3 mediated effects. Despite these findings, several aspects remain to be critically discussed. While Kim et al. reported increased TIM-3 expression in the colonic mucosa of CD patients, contrary effects were found in isolated PBMCs by the same group and additionally studies from Shi et al. [37, 38]. Ambivalent TIM-3 expression patterns were also described in context with other disorders. T cells from patients with allergic asthma or chronic hepatitis C infection expressed elevated levels of TIM-3, whereas sarcoidosis patients showed downregulated levels of TIM-3 in their lungs [42–44].

**Fig. 1:**
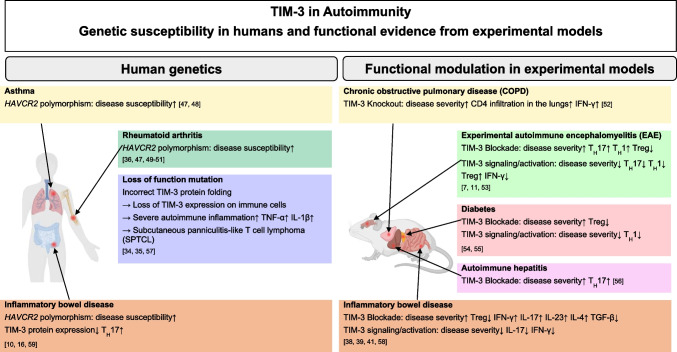
TIM-3 in Autoimmunity Genetic susceptibility in humans and functional evidence from experimental models. *HAVCR2* polymorphisms in humans overall increase the susceptibility for diseases such as asthma, rheumatoid arthritis, or IBD (*left*). In in vivo experimental models, blocking TIM-3 is associated with increased disease severity in COPD, EAE, diabetes, autoimmune hepatitis or IBD. Contrarily, TIM-3 signaling activation can ameliorate disease severity (*right*) [7, 10, 11, 16, 34, 35, 36, 38, 39, 41, 47, 48, 49, 50, 51, 52, 53, 54, 55, 56, 57, 58, 59]

In summary, TIM-3 expression and function are inconsistent and can vary depending on the tissue and disease context, contributing to the ongoing debate whether TIM-3 acts as a co-inhibitory or co-stimulatory receptor. A few studies have reported that TIM-3 can promote inflammatory processes, function as a co-stimulatory receptor, and enhance TCR signaling under specific conditions [12, 20, 45, 46]. Lee et al. described an in vitro setting in which murine CD4^+^T cells under Th1 stimulation and infected with retrovirus encoding TIM-3 initially can augment T-cell activation and increase IFN-γ production. However, cross-linking TIM-3 with an agonistic antibody rapidly inhibited T-cell activation [20]. Comparable effects were reported by Avery and colleagues. In their study, enforced TIM-3 expression was accompanied by enhanced TCR signaling in primary T cells and elevated levels of short-lived effector cells [12]. Further in vitro models suggested TIM-3-mediated co-stimulation via MEK-ERK or Akt-mTOR signaling [45] and a recently published study from Alamir et al. described TIM-3 as an inhibitory receptor on spheroid-suppressed cytotoxic T lymphocytes but not on active cells in a two-dimensional tissue culture model. [46]. Taken together, this underlined that TIM-3’s role is ambivalent highly depending on the functional context model applied. On the other hand, findings from *HAVCR2* polymorphisms and modulation experiments in autoimmune diseases and IBD, using TIM-3 with blocking antibodies or activation by its ligand galectin-9, rather indicate a predominant inhibitory role, particularly in colitis (Fig. 1) [34, 35, 38, 39, 41].

## Intracellular signaling mediated by TIM-3 and the connection to TCR signaling

**Fig. 2:**
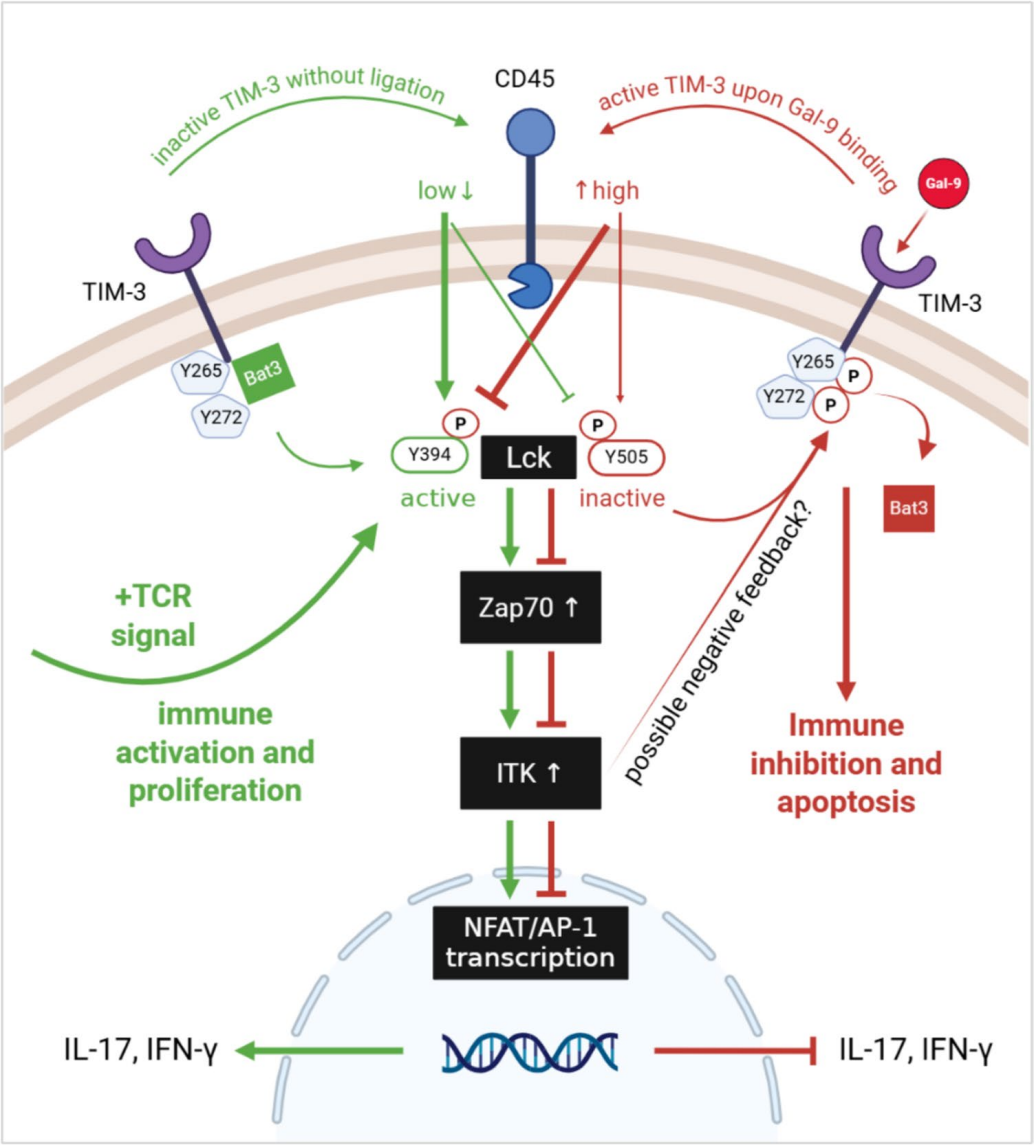
Proposed schematic interplay of TIM-3 with TCR signaling. Upon stimulation of TIM-3 by Galectin-9 (red pathway), increased CD45 expression leads to dephosphorylation of the activating tyrosine (Y394) on Lck, reducing its kinase activity and suppressing downstream TCR signaling. Concurrently, Gal-9 binding induces phosphorylation of the intracellular tyrosines Y265 and Y272 on TIM-3 and displacement of the inhibitory adaptor Bat3. This process inhibits NFAT/AP-1-driven transcription and promotes T cell exhaustion or apoptosis. ITK may further enhance this phosphorylation, potentially creating a negative feedback loop to attenuate T cell activation. Conversely, in the absence of Gal-9 (green pathway), TIM-3 remains unphosphorylated and retains Bat3, which supports Lck function and TCR signaling. Low CD45 levels enhance Y394 phosphorylation on Lck and maintain its activity, promoting downstream signaling through Zap70 and ITK, ultimately leading to NFAT/AP-1 activation and T cell proliferation. Without TIM-3 activation, the production of IFN-γ and IL-17 cytokines is generally preserved, whereas activation of TIM-3 by Gal-9 suppresses IFN-γ and IL-17 cytokine production, as postulated in experimental models (see also Fig. 1)

As TIM-3 can regulate T cell activation, its proximal connection to TCR signaling will be explained in more detail. Intracellular motifs of the TCR need to be phosphorylated by the lymphocyte cell-specific protein-tyrosine kinase (Lck) or on less account by the Src family tyrosine kinase Fyn, in order to activate TCR signaling. Crucial for this phosphorylation step is the location of Lck in proximity to the cell membrane [60]. TIM-3 can mediate the migration of active Lck towards the cell membrane. In the absence of a TIM-3 ligand, the human leukocyte antigen B (HLA-B)-associated transcript 3 (Bat3) is linked to two out of five tyrosine residues (Y265 and Y272) on the intracellular tail of TIM-3 which facilitates Lck recruitment and can contribute to TCR signaling [14, 15, 61, 62]. Contrarily, upon binding of TIM-3’s ligand Galectin-9, Y265 and Y272 tyrosine residues can get phosphorylated either by IL2-inducible tyrosine kinase (ITK) or Src family kinase Lck [15]. As a consequence, Bat3 initiates the inactivation of Lck via the C-terminal Src kinase CsK, that phosphorylates the inhibitory site of Lck [20, 61]. Subsequently, TCR signaling can be interrupted and cell proliferation inhibited due to TIM-3 activation [61]. Simultaneously, stimulation of TIM-3 via Gal-9 can upregulate the expression of CD45 which can facilitate the dephosphorylation of the active form of Lck thereby diminish Lck activity and dampening TCR signaling [63]. Taken together, the TIM-3 receptor contributes to TCR signaling when it is unbound by a ligand and its intracellular tyrosine residues are linked to Bat3. Conversely, TIM-3 can dampen TCR signaling when a ligand is bound and the tyrosine residues of the intracellular tail are phosphorylated by Lck or ITK (Fig. 2) [20]. The ligand-dependent loss of Bat3 from TIM-3 and the ITK-mediated phosphorylation of its intracellular tyrosines together indicate how TIM-3 can influence early steps of TCR signaling.

Moving to the further downstream events, the TCR signaling cascade involves the binding of zeta-chain-associated protein kinase 70 (Zap-70), a key step that allows Lck to phosphorylate and activate Zap-70. Subsequently, phospholipase C gamma 1 (PLCγ1) gets activated by ITK and the 2nd messengers namely inositol trisphosphate (IP3) and diacylglycerol (DAG) are produced that initiate the translocation of the transcription factors nuclear factor of activated T cells (NFAT), nuclear factor κB (NFκB), and activator protein-1 (AP-1) in the nucleus. Subsequently, cell proliferation- and activation-associated genes get transcribed, which is accompanied by cytokine release [60]. In context with TIM-3, it was reported that TIM-3 can limit the available pools of Lck and PLCγ1 upon stimulation with anti-CD3/anti-CD28 antibodies leading to interrupted TCR signaling [64]. Additionally, Lee et al. demonstrated a dependence of ZAP70 and the adaptor protein SLP-76 for TIM-3-mediated TCR activation [20]. Moreover, point mutations within the intracellular tyrosine residues of the TIM-3 receptor were found to restore NFAT activity [64]. The cytoplasmic tail of TIM-3 is also required for the suppression of both IL-2 production and AP-1/NFAT activation [65], suggesting that these residues are central for the immunoinhibitory signaling of TIM-3. In summary, evidence from several studies suggests that TIM-3 modulates TCR signaling by interacting with it in different steps of the cascade.

## Open questions in the TIM-3 signaling pathway: what role does ITK play?

**Fig. 3:**
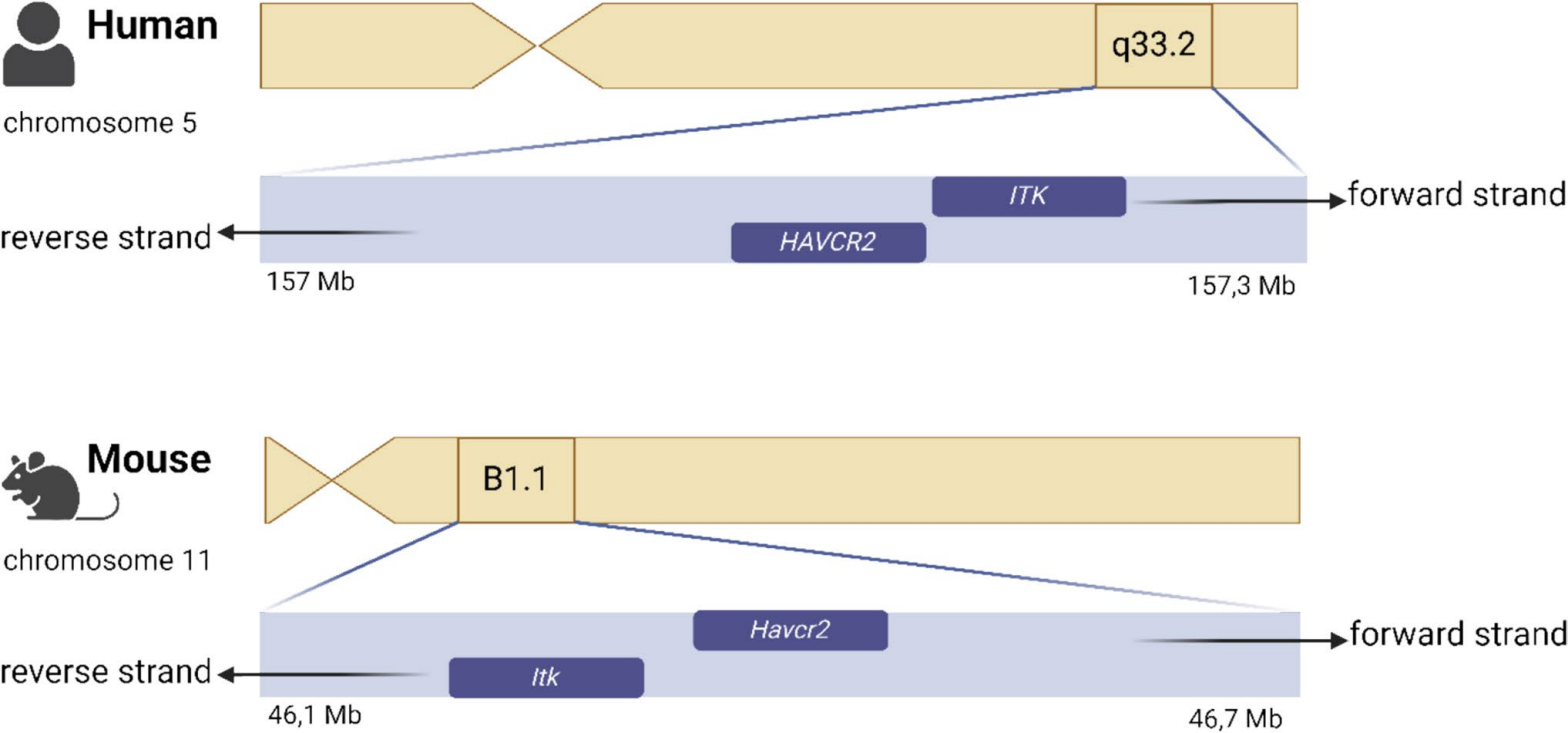
Genomic location of the genes for TIM-3 and ITK. HAVCR2 and ITK are located on chromosome 5.q33 in humans and chromosome 11B1 in mice respectively. A region known for its association with the development of autoimmune diseases. Abbreviations: Mb (Megabases)

TIM-3 signaling is closely linked to TCR signaling and both pathways are shown to influence each other. As described above, interactions of TIM-3 with Lck, Fyn, ITK, Zap70, or PLCγ1 were reported. Off note, the corresponding genes *HAVCR2, Lck, Fyn Zap70,* or *PLCG1* are all located on different chromosomes, except for *HAVCR2* and *ITK* [18]. Both *HAVCR2* and *ITK* are located on chromosome 5, cytoband q33.3, in close proximity (Fig. 3). Interestingly, the gene region on chromosome 5.q33 is also the home of T_H_2 cytokines, which are frequently associated with autoimmune diseases in humans [66]. Not only TIM-3 but also ITK is involved in the development and maintenance of autoimmune diseases, like asthma and multiple sclerosis, or inflammatory bowel disease [10, 36, 38, 41, 67, 68]. ITK can phosphorylate the intracellular tyrosine residues of TIM-3 in HEK293T cells [15]. Phosphorylation of these tyrosine residues reportedly leads to apoptosis of T_H_1 cells and a downregulation of the T_H_1 immune response [15]. Based on the considerations outlined above, the involvement of ITK in TIM-3 signal transduction appears to be of particular interest. While the roles of Lck and Bat3 in TIM-3 signaling are well characterized, the contribution of ITK remains insufficiently understood but might act as an important regulatory node in the TCR signaling network balancing activation and exhaustion. The question arises why ITK, in particular, is capable of phosphorylating the tyrosine residues of the intracellular tail and underscores the need for further investigation.

Several questions remain unanswered about the TIM-3/ITK connection in this regard. Notably, among T-helper cell types, T_H_2 cells exhibit the highest levels of ITK, while highest levels of TIM-3 are found on T_H_1 cells [7, 67, 68]. In contrast, T_H_1 cells display lower ITK activity compared to other T_H_ cell subtypes [69, 70]. The phosphorylation of the intracellular tail of TIM-3 by ITK and the subsequent apoptosis could also be the reason for low ITK expression levels in T_H_1 cells, as T_H_1 cells with elevated ITK expression levels are more likely to go into apoptosis. It remains to be clarified in more detail if phosphorylation of the intracellular tail of TIM-3 by ITK in T_H_1 cells induces apoptosis in a direct ITK-dependent manner. A direct interaction between TIM-3 and ITK, or parallel to the interaction between LCK and TCR signal transduction, is an interesting question that cannot be fully answered based on the currently available data. In our opinion, there is a neglected connection between TIM-3 and ITK, or rather an interplay whose investigation could clarify the exact molecular downstream signaling of TIM-3. Further elucidation of these circumstances through more in-depth experiments, particularly in the context of the individual T_H_ cell subtypes, is necessary.

## Conclusion

TIM-3 is an important immune checkpoint receptor and a promising target in cancer and autoimmune therapies. The underlying signaling mechanisms remain only partially elucidated, although several studies have demonstrated links to TCR signaling, as summarized in Table 1. Notably, TIM-3 signaling appears to be interconnected with TCR signaling, since TCR-associated kinases such as Lck, Fyn, and ITK can modulate the tyrosine residues of the intracellular tail of TIM-3, conversely, TIM-3 can influence TCR signaling. In context with autoimmunity and inflammatory disorders like IBD, the linkage of TIM-3 and ITK is interesting even though insufficiently investigated. Their close proximity on the same chromosome and their implied direct interaction is a promising connection that should be examined more closely in future research approaches.

**Table 1:** Interactions of TIM-3 with the TCR signaling pathway

Interaction described	Explanation	Ref
LCK → TIM-3 TIM-3 → PI3K	Phosphorylation of tyrosines 256/263 of TIM-3 at the intracellular tail and recruitment of p85 PI3K is necessary for TIM-3 downstream signaling to NFAT/AP-1 and NF-kB. Src family kinases LCK and FYN can phosphorylate TIM-3. The dependence of src family kinases and ZAP70 and SLP76 proteins in TIM-3 downstream signaling indicates a proximity to the TCR signaling pathway	[20]
TIM-3 → LCK	Upon binding of Gal-9, increased levels of CD45 are detected leading to diminished LCK activity by dephosphorylation of the active form of LCK (pY394) and dampening TCR signaling	[63]
ITK → TIM-3	ITK phosphorylates TIM-3 in HEK293T cells at Y265 of the cytoplasmic tail. Interaction of Gal-9 with TIM-3 leads to Y265 phosphorylation, resulting in impaired TCR signaling	[15]
TIM-3 → LCK and PLCγ1 TIM-3 → NF-kB/NFAT activity	Tyrosine residues Y265/Y272 at the cytoplasmic tail of TIM-3 are critical for the function. TIM-3 interrupts downstream signals originating from TCR by inhibiting NF-kB/NFAT activation and IL-2 expression. TCR stimulation enables association of Src kinase Lck, and PLCg with TIM-3. TIM-3 inhibits LCK and PLCg, limiting them to carry out the activation steps required for TCR signaling	[64]
TIM-3 → PI3K	TCR stimulation with αCD3/CD28 as well as γc cytokines IL-2, IL-7- IL-15 and IL-21 drives TIM-3 expression on CD4^+^/CD8^+^ T cells in vitro. TIM-3^+^ T cells are more sensitive to apoptosis through galectin-9. Blocking the PI3K pathway by using a PI3K inhibitor diminished TIM-3 expression	[71]
TIM-3 → NFAT/AP-1	TIM-3 expression reduced the stimulation-induced dephosphorylation and nuclear translocation of NFAT. The cytoplasmic tail was necessary for IL-2 suppression as well as AP-1 and NFAT activation. T cell IL-2 production may be suppressed by TIM-3, leading to reduction of NFAT dephosphorylation and AP-1 transcription	[65]

## Data Availability

Not applicable.

## References

[1] Marshall JS et al (2018) An introduction to immunology and immunopathology. Allergy Asthma Clin Immunol 14(Suppl 2):49 30263032 10.1186/s13223-018-0278-1 PMC6156898

[2] Gorentla BK, Zhong XP (2012) T cell receptor signal transduction in T lymphocytes. J Clin Cell Immunol 2012(Suppl 12):5 23946894 10.4172/2155-9899.S12-005 PMC3740441

[3] Cantrell DA (2002) T-cell antigen receptor signal transduction. Immunology 105(4):369–374 11985657 10.1046/j.1365-2567.2002.01391.x PMC1782684

[4] Courtney AH, Lo WL, Weiss A (2018) TCR signaling: mechanisms of initiation and propagation. Trends Biochem Sci 43(2):108–123 29269020 10.1016/j.tibs.2017.11.008 PMC5801066

[5] Chen L, Flies DB (2013) Molecular mechanisms of T cell co-stimulation and co-inhibition. Nat Rev Immunol 13(4):227–242 23470321 10.1038/nri3405 PMC3786574

[6] Joller N, Anderson AC, Kuchroo VK (2024) LAG-3, TIM-3, and TIGIT: distinct functions in immune regulation. Immunity 57(2):206–222 38354701 10.1016/j.immuni.2024.01.010 PMC10919259

[7] Monney L et al (2002) Th1-specific cell surface protein Tim-3 regulates macrophage activation and severity of an autoimmune disease. Nature 415(6871):536–541 11823861 10.1038/415536a

[8] Wu M et al (2023) Identification of a novel small-molecule inhibitor targeting TIM-3 for cancer immunotherapy. Biochem Pharmacol 212:115583 37148978 10.1016/j.bcp.2023.115583

[9] Acharya N, Sabatos-Peyton C, Anderson AC (2020) Tim-3 finds its place in the cancer immunotherapy landscape. J Immunother Cancer. 10.1136/jitc-2020-000911 32601081 10.1136/jitc-2020-000911 PMC7326247

[10] Baldrich A et al (2024) Post-transplant inflammatory bowel disease associated with donor-derived TIM-3 deficiency. J Clin Immunol 44(3):1–10 10.1007/s10875-024-01667-z PMC10873237 38363399

[11] Zhu C et al (2005) The Tim-3 ligand galectin-9 negatively regulates T helper type 1 immunity. Nat Immunol 6(12):1245–1252 16286920 10.1038/ni1271

[12] Avery L et al (2018) Tim-3 co-stimulation promotes short-lived effector T cells, restricts memory precursors, and is dispensable for T cell exhaustion. Proc Natl Acad Sci U S A 115(10):2455–2460 29463725 10.1073/pnas.1712107115 PMC5877951

[13] Wolf Y, Anderson AC, Kuchroo VK (2020) TIM3 comes of age as an inhibitory receptor. Nat Rev Immunol 20(3):173–185 31676858 10.1038/s41577-019-0224-6 PMC7327798

[14] Rangachari M et al (2012) Bat3 promotes T cell responses and autoimmunity by repressing Tim-3–mediated cell death and exhaustion. Nat Med 18(9):1394–1400 22863785 10.1038/nm.2871 PMC3491118

[15] van de Weyer PS et al (2006) A highly conserved tyrosine of Tim-3 is phosphorylated upon stimulation by its ligand galectin-9. Biochem Biophys Res Commun 351(2):571–576 17069754 10.1016/j.bbrc.2006.10.079

[16] Huang Y-H et al (2015) *CEACAM1 regulates TIM-3-mediated tolerance and exhaustion.* Nature **517**(7534): p. 386–390. corrigendum: 10.1038/nature17421 10.1038/nature13848 PMC4297519 25363763

[17] Consortium TU (2022) *UniProt: the Universal Protein Knowledgebase in 2023.* Nucleic Acids Res 51(D1): p. D523–D531. 10.1093/nar/gkac1052 PMC9825514 36408920

[18] Uhlen M et al (2015) Tissue-based map of the human proteome. Science. 10.1126/science.1260419 25613900 10.1126/science.1260419

[19] Anderson AC, Xiao S, Kuchroo VK (2007) Tim protein structures reveal a unique face for ligand binding. Immunity 26(3):273–275 17376389 10.1016/j.immuni.2007.03.004 PMC9903193

[20] Lee J et al (2011) Phosphotyrosine-dependent coupling of Tim-3 to T-cell receptor signaling pathways. Mol Cell Biol 31(19):3963–3974 21807895 10.1128/MCB.05297-11 PMC3187355

[21] Cao E et al (2007) T cell immunoglobulin mucin-3 crystal structure reveals a galectin-9-independent ligand-binding surface. Immunity 26(3):311–321 17363302 10.1016/j.immuni.2007.01.016

[22] Kuchroo VK et al (2006) TIM family of genes in immunity and tolerance. Adv Immunol 91(91):227–249 16938542 10.1016/S0065-2776(06)91006-2

[23] Lv Y, Ma X, Ma Y, Du Y, Feng J. (2022) A new emerging target in cancer immunotherapy: Galectin-9 (LGALS9). Genes Dis 10(6):2366-2382. 10.1016/j.gendis.2022.05.020 10.1016/j.gendis.2022.05.020 PMC10404877 37554219

[24] Yang R et al (2022) Development and characterization of anti-galectin-9 antibodies that protect T cells from galectin-9-induced cell death. J Biol Chem. 10.1016/j.jbc.2022.101821 35283189 10.1016/j.jbc.2022.101821 PMC9006662

[25] Nagata S et al (2016) Exposure of phosphatidylserine on the cell surface. Cell Death Differ 23(6):952–961 26891692 10.1038/cdd.2016.7 PMC4987739

[26] Santiago C et al (2007) Structures of T cell immunoglobulin mucin protein 4 show a metal-ion-dependent ligand binding site where phosphatidylserine binds. Immunity 27(6):941–951 18083575 10.1016/j.immuni.2007.11.008 PMC2330274

[27] Fadok VA et al (1992) Exposure of phosphatidylserine on the surface of apoptotic lymphocytes triggers specific recognition and removal by macrophages. J Immunol 148(7):2207–2216 1545126

[28] DeKruyff RH et al (2010) T cell/transmembrane, Ig, and mucin-3 allelic variants differentially recognize phosphatidylserine and mediate phagocytosis of apoptotic cells. J Immunol 184(4):1918–1930 20083673 10.4049/jimmunol.0903059 PMC3128800

[29] Avgousti DC et al (2016) A core viral protein binds host nucleosomes to sequester immune danger signals. Nature 535(7610):173–177 27362237 10.1038/nature18317 PMC4950998

[30] Chiba S et al (2012) Tumor-infiltrating DCs suppress nucleic acid-mediated innate immune responses through interactions between the receptor TIM-3 and the alarmin HMGB1. Nat Immunol 13(9):832–842 22842346 10.1038/ni.2376 PMC3622453

[31] Dolina JS, Braciale TJ, Hahn YS (2014) Liver-primed CD8+ T cells suppress antiviral adaptive immunity through galectin-9-independent T-cell immunoglobulin and mucin 3 engagement of high-mobility group box 1 in mice. Hepatology 59(4):1351–1365 24677194 10.1002/hep.26938 PMC3970181

[32] Gandhi AK et al (2024) Structural aspects of CEACAM1 interactions. Eur J Clin Invest 54(Suppl 2):e14357 39555955 10.1111/eci.14357 PMC12938038

[33] Dankner M et al (2017) CEACAM1 as a multi-purpose target for cancer immunotherapy. Oncoimmunology 6(7):e1328336 28811966 10.1080/2162402X.2017.1328336 PMC5543821

[34] Gayden T et al (2018) Germline HAVCR2 mutations altering TIM-3 characterize subcutaneous panniculitis-like T cell lymphomas with hemophagocytic lymphohistiocytic syndrome. Nat Genet 50(12):1650–1657 30374066 10.1038/s41588-018-0251-4

[35] Polprasert C et al (2019) Frequent germline mutations of HAVCR2 in sporadic subcutaneous panniculitis-like T-cell lymphoma. Blood Adv 3(4):588–595 30792187 10.1182/bloodadvances.2018028340 PMC6391671

[36] Zhang R et al (2019) Association between T-cell immunoglobulin and mucin domain 3 (TIM-3) genetic polymorphisms and susceptibility to autoimmune diseases. Immunol Invest 48(6):563–576 31044630 10.1080/08820139.2019.1599009

[37] Kim MJ, Lee WY, Choe YH (2014) Expression of TIM-3, human β-defensin-2, and FOXP3 and correlation with disease activity in pediatric Crohn’s disease with infliximab therapy. Gut Liver 9(3):370 10.5009/gnl13408 PMC4413971 25071071

[38] Shi F et al (2012) Dysregulated Tim-3 expression and its correlation with imbalanced CD4 helper T cell function in ulcerative colitis. Clin Immunol 145(3):230–240 23117395 10.1016/j.clim.2012.09.001

[39] Li X et al (2010) Involvement of T cell Ig mucin-3 (Tim-3) in the negative regulation of inflammatory bowel disease. Clin Immunol 134(2):169–177 19913460 10.1016/j.clim.2009.09.012

[40] Gautron AS et al (2014) Enhanced suppressor function of TIM-3+ FoxP3+ regulatory T cells. Eur J Immunol 44(9):2703–2711 24838857 10.1002/eji.201344392 PMC4165702

[41] Xiong H et al (2023) Effect of exogenous galectin-9, a natural TIM-3 ligand, on the severity of TNBS-and DSS-induced colitis in mice. Int Immunopharmacol 115:109645 36610329 10.1016/j.intimp.2022.109645

[42] Tang F et al (2015) Upregulation of Tim-3 on CD4^+^T cells is associated with Th1/Th2 imbalance in patients with allergic asthma. Int J Clin Exp Med 8(3):3809 26064278 PMC4443112

[43] Golden-Mason L et al (2009) Negative immune regulator Tim-3 is overexpressed on T cells in hepatitis C virus infection and its blockade rescues dysfunctional CD4+ and CD8+ T cells. J Virol 83(18):9122–9130 19587053 10.1128/JVI.00639-09 PMC2738247

[44] Idali F et al (2009) Altered expression of T cell immunoglobulin-mucin (TIM) molecules in bronchoalveolar lavage CD4^+^T cells in sarcoidosis. Respir Res 10(1):42 19480659 10.1186/1465-9921-10-42 PMC2694180

[45] Kataoka S et al (2021) The costimulatory activity of Tim-3 requires Akt and MAPK signaling and its recruitment to the immune synapse. Sci Signal 14(687):eaba0717 34131021 10.1126/scisignal.aba0717 PMC9741863

[46] Alamir H et al (2025) TIM3 is a context-dependent coregulator of cytotoxic T cell function. Sci Signal 18(905):eadk4594 40986642 10.1126/scisignal.adk4594

[47] Chae SC et al (2004) The association of TIM-3 gene polymorphism with atopic disease in Korean population. Hum Immunol 65(12):1427–1431 15603868 10.1016/j.humimm.2004.07.002

[48] Wu Q-W et al (2009) Family-based association study of Tim-1 and Tim-3 gene polymorphisms with childhood asthma in Chinese trios. Int Arch Allergy Immunol 150(3):252–260 19494522 10.1159/000222677

[49] Song YW et al (2011) T-cell immunoglobulin and mucin domain 3 genetic polymorphisms are associated with rheumatoid arthritis independent of a shared epitope status. Hum Immunol 72(8):652–655 21565238 10.1016/j.humimm.2011.04.007

[50] Xu J et al (2011) The− 1541 C> T and+ 4259 G> T of TIM-3 polymorphisms are associated with rheumatoid arthritis susceptibility in a Chinese Hui population. Int J Immunogenet 38(6):513–518 21989170 10.1111/j.1744-313X.2011.01046.x

[51] Liu R et al (2018) TIM-3 rs1036199 polymorphism increases susceptibility to autoimmune diseases: evidence based on 4200 subjects. Biosci Rep 38(6):BSR20181235 30377229 10.1042/BSR20181235 PMC6250810

[52] Ke J et al (2024) Nfil3/Tim3 axis regulates effector Th1 inflammation in COPD mice. Front Immunol 15:1482213 39555065 10.3389/fimmu.2024.1482213 PMC11563780

[53] Tang L et al (2022) Tim-3 relieves experimental autoimmune encephalomyelitis by suppressing MHC-II. Front Immunol 12:770402 35095844 10.3389/fimmu.2021.770402 PMC8793033

[54] Kanzaki M et al (2012) Galectin-9 and T cell immunoglobulin mucin-3 pathway is a therapeutic target for type 1 diabetes. Endocrinology 153(2):612–620 22186414 10.1210/en.2011-1579

[55] Sánchez-Fueyo A et al (2003) Tim-3 inhibits T helper type 1–mediated auto-and alloimmune responses and promotes immunological tolerance. Nat Immunol 4(11):1093–1101 14556005 10.1038/ni987

[56] Wu H et al (2021) Tim-3 suppresses autoimmune hepatitis via the p38/MKP-1 pathway in Th17 cells. FEBS Open Bio 11(5):1406–1416 33728805 10.1002/2211-5463.13148 PMC8091815

[57] Wegehaupt O et al (2020) TIM-3 deficiency presenting with two clonally unrelated episodes of mesenteric and subcutaneous panniculitis-like T-cell lymphoma and hemophagocytic lymphohistiocytosis. Pediatr Blood Cancer 67(6):e28302 32285995 10.1002/pbc.28302

[58] Wang F et al (2024) Macrophage Tim-3 maintains intestinal homeostasis in DSS-induced colitis by suppressing neutrophil necroptosis. Redox Biol. 10.1016/j.redox.2024.103072 38330550 10.1016/j.redox.2024.103072 PMC10865407

[59] Lin D et al (2023) Association of TIM-3 with anterior uveitis and associated systemic immune diseases: a Mendelian randomization analysis. Front Med 10:1183326 10.3389/fmed.2023.1183326 PMC10313383 37396905

[60] Shah K et al (2021) T cell receptor (TCR) signaling in health and disease. Signal Transduct Target Ther 6(1):412 34897277 10.1038/s41392-021-00823-w PMC8666445

[61] Yan Z et al (2025) TIM-3 teams up with PD-1 in cancer immunotherapy: mechanisms and perspectives. Mol Biomed 6(1):27 40332725 10.1186/s43556-025-00267-6 PMC12058639

[62] Kandel S et al (2021) The TIM3/Gal9 signaling pathway: an emerging target for cancer immunotherapy. Cancer Lett 510:67–78 33895262 10.1016/j.canlet.2021.04.011 PMC8168453

[63] Clayton KL et al (2014) T cell Ig and mucin domain-containing protein 3 is recruited to the immune synapse, disrupts stable synapse formation, and associates with receptor phosphatases. J Immunol 192(2):782–791 24337741 10.4049/jimmunol.1302663 PMC4214929

[64] Tomkowicz B et al (2015) TIM-3 suppresses anti-CD3/CD28-induced TCR activation and IL-2 expression through the NFAT signaling pathway. PLoS ONE 10(10):e0140694 26492563 10.1371/journal.pone.0140694 PMC4619610

[65] Lee MJ et al (2012) Down-regulation of interleukin-2 production by CD4^+^T cells expressing TIM-3 through suppression of NFAT dephosphorylation and AP-1 transcription. Immunobiology 217(10):986–995 22445722 10.1016/j.imbio.2012.01.012

[66] McIntire JJ et al (2001) Identification of (an airway hyperreactivity regulatory locus) and the linked gene family. Nat Immunol 2(12):1109–1116 11725301 10.1038/ni739

[67] Lechner KS, Neurath MF, Weigmann B (2020) Role of the IL-2 inducible tyrosine kinase ITK and its inhibitors in disease pathogenesis. J Mol Med 98:1385–1395 32808093 10.1007/s00109-020-01958-z PMC7524833

[68] Lechner K et al (2021) Targeting of the Tec kinase ITK drives resolution of T cell-mediated colitis and emerges as potential therapeutic option in ulcerative colitis. Gastroenterology 161(4):1270 34224738 10.1053/j.gastro.2021.06.072

[69] Kannan A et al (2015) Allele-sensitive mutant, Itkas, reveals that Itk kinase activity is required for Th1, Th2, Th17, and iNKT-cell cytokine production. Eur J Immunol 45(8):2276–2285 25989458 10.1002/eji.201445087 PMC5730406

[70] Zhong Y et al (2014) Targeting interleukin-2-inducible T-cell kinase (ITK) in T-cell related diseases. Postdoc J 2(6):1–11 27917390 10.14304/surya.jpr.v2n6.1 PMC5134889

[71] Mujib S et al (2012) Antigen-independent induction of Tim-3 expression on human T cells by the common γ-chain cytokines IL-2, IL-7, IL-15, and IL-21 is associated with proliferation and is dependent on the phosphoinositide 3-kinase pathway. J Immunol 188(8):3745–3756 22422881 10.4049/jimmunol.1102609

